# Routine stress testing with subsequent coronary angiography versus standard of care in high-risk patients after percutaneous coronary intervention: An updated *meta*-analysis of randomized controlled trials

**DOI:** 10.1016/j.ijcha.2025.101681

**Published:** 2025-04-12

**Authors:** Umar G. Adamu, David M. Mashilo, Anupa Patel, Nqoba Tsabedze

**Affiliations:** Division of Cardiology, Department of Internal Medicine, School of Clinical Medicine, Faculty of Health Sciences, University of the Witwatersrand, Johannesburg, South Africa

**Keywords:** Percutaneous coronary intervention, Functional testing, Standard of care, High-risk patients, Target lesion revascularization, Myocardial infarction

## Abstract

Routine functional stress testing with subsequent coronary angiography is undertaking to detect early restenosis and guide revascularization in high-risk patients after percutaneous coronary intervention (PCI). However, the safety and efficacy of routine functional stress testing over standard care is still debatable. This *meta*-analysis compares routine functional stress testing vs. standard care in high-risk patients after PCI. We systematically searched PubMed, Embase, and Cochrane Central databases to identify randomized controlled trials (RCTs) that compared functional stress testing versus standard of care after PCI in high-risk patients from inception to January 2025. We calculated the risk ratios (RRs) with 95 % confidence intervals (CIs) using the random-effects model for clinical outcomes. Four RCTs with 6,290 patients, of whom 3,206 (51 %) underwent routine functional stress testing were included in our analyses. The incidence of target lesion revascularization (TLR) was higher in routine functional stress testing (RR: 1.49; 95 % CI: 1.02–2.18; p = 0.038) compared with standard care. However, no statistically significant difference was observed for individual outcomes of all-cause mortality (RR: 0.89; 95 % CI: 0.48–1.18; p = 0.198), myocardial infarction (MI) (RR: 0.62; 95 % CI: 0.31–1.24; p = 0.174), and hospitalization for any cause (RR: 1.22; 95 % CI: 0.24–6.10; p = 0.809). The risk of MACE did not significantly differ between the groups (RR: 1.11; 95 % CI: 0.82–1.51; p = 0.480). Routine functional stress testing after PCI in high-risk patients was associated with increased incidence of target lesion revascularization, without reducing the risk of major adverse cardiovascular events.

## Highlights

• This study compared routine functional stress testing with standard care for high-risk patients after PCI.
• Routine stress testing is associated with an increased risk of target lesion revascularization (TLR).
• No significant differences were observed in all-cause mortality or myocardial infarction rates between the groups.
• Routine stress testing did not reduce major adverse cardiovascular events (MACE).
• These findings suggest that post-PCI coronary angiography should be reserved for patients with recurrent or new symptoms.

## Graphical abstract

ACM, All-cause mortality; MACE, Major adverse cardiovascular events; MI, Myocardial infarction; PCI, Percutaneous coronary intervention; RCTs, Randomized controlled trials; TLR, Target lesion revascularisation.

**Figure:**
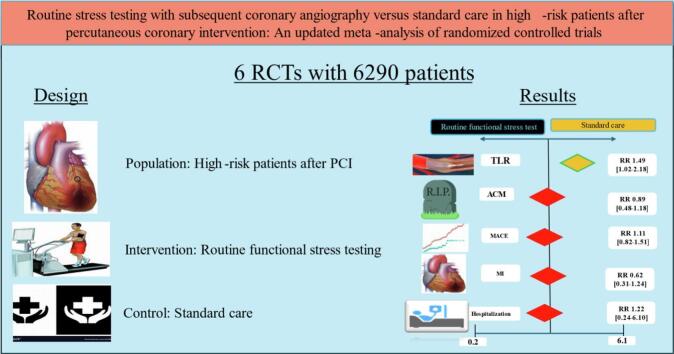

## 1 Introduction

Percutaneous coronary intervention (PCI) has become a cornerstone of the management of coronary artery disease, particularly in high-risk patients with complex lesions or multiple comorbidities [1]. Functional stress testing with coronary angiography is an established surveillance modality in patients with high-risk anatomical features or clinical characteristics after PCI who are symptomatic and to assess residual ischaemia [2,3]. However, the optimal follow-up strategy for asymptomatic patients post-PCI remains a subject of debate.

Previous studies have reported substantial heterogeneity in follow-up strategies among hospitals and interventional cardiologists, particularly among those with a high frequency of stress testing [4]. In a recent observational survey in US Veterans Affairs hospitals, the proportion of stress tests consistent with obstructive coronary artery disease (CAD) was similar between symptomatic and asymptomatic patients, with minimal site variation [5]. The recent 2023 American guidelines on revascularisation gave a Class III recommendation for functional stress testing in asymptomatic patients [2]. However, the 2024 European Society of Cardiology guidelines for the management of chronic coronary syndromes noted recent evidence but failed short of recommending a specific class for its use in this group of patients [3]. Therefore, there is still lack of consensus as to the significance of surveillance stress testing in high-risk PCI.

Since the publication of the only *meta*-analysis evaluating the role of routine follow-up coronary angiography after PCI [6] subsequent studies have been published, including randomized data [7] and several post-hoc analyses [[8], [9], [10]]. Therefore, we aimed to perform an updated systematic review and *meta*-analysis of RCTs to investigate the relevance of routine functional stress testing versus standard care in high-risk patients after PCI, exploring populations with diabetes, type of stent used, and follow-up.

## 2 Methods

This systematic review and *meta*-analysis was performed in accordance with the Cochrane Collaboration Handbook for Systematic Reviews of Interventions and the Preferred Reporting Items for Systematic Reviews and Meta-analysis (PRISMA) statement [11]. The analysis was prospectively registered in the PROSPERO International Prospective Register of Systematic Reviews (CRD42025639900). Institutional board approval was not required as the data used in this study were publicly available, and with prior institutional review board approval.

### 2.1 Literature search strategy

PubMed, Embase, and Cochrane Central Register of Controlled Trials were systematically searched from inception to January 2025. We used the Boolean operators-based PRISMA search strategy using patient, intervention, comparison, and outcomes (PICO) format. The following key words were used for the search: ‘high-risk’, ‘multivessel disease’, ‘elderly’, ‘diabetes’, ‘percutaneous coronary intervention’, ‘PCI’. The full search strategy for each database is provided in Table S1. All the identified articles were assessed using the inclusion and exclusion criteria. Additional articles were identified by snowballing of the references of previous RCTs and *meta*-analysis. In cases of overlapping studies, the one with most participants was included in the *meta*-analysis.

### 2.2 Study selection and eligibility criteria

All retrieved articles from each of the databases were exported to Rayyan reference manager (Cambridge, MA, USA) and duplicates were removed. Two reviewers (UGA and DM) independently performed the initial screening of the remaining articles using the titles and abstract following predefined search criteria. Disagreements between authors were resolved by consensus or by discussion with the other reviewers (AP and NT). We included studies that met the following eligibility criteria: (1) RCTs; (2) compared routine functional stress testing to standard care; (3) patients who underwent high-risk PCI; (4) reporting at least one outcomes of interest. We excluded studies with (1) no control group; (2) no outcomes of interest; and (3) with an overlapping population. There were no restrictions concerning the date, language of publication and no filters based on year of publication, author name, and institution/country of publication.

The outcomes of interest included all-cause mortality, myocardial infarction (MI), combined all-cause mortality and MI, and target lesion revascularisation. We also included the composite endpoints of major adverse cardiovascular event (MACE). The definition of MACE varied slightly across the included studies and is provided in Table S2.

### 2.3 Data extraction and quality assessment

Two reviewers (UGA and DM) independently extracted the data from the shortlisted articles following predefined search criteria. Disagreements between authors were resolved by consensus or by discussion with the other reviewers (AP and NT). The extracted data included author’s first name, year of publication, name and type of the study, sample size, demographics of the patients like mean age, gender, and comorbidities including hypertension, diabetes, and previous myocardial infarction, and the duration of follow-up. We evaluated the risk of bias in the included RCTs using version 2 of the Cochrane Risk of Bias Assessment Tool (RoB 2) [12]. The results of the risk of bias assessment were plotted using the robvis online tool. Two authors (UGA and DM) independently conducted the risk of bias assessment. Disagreements were resolved by consensus after discussing the reasons for the discrepancies.

### 2.4 Statistical analysis

All outcomes were assessed on an intention-to-treat basis. Risk ratios (RRs) with 95 % confidence intervals (CIs) were pooled with Mantel-Haenszel method. A random-effects *meta*-analysis was performed for all binary endpoints and forest plots were generated. We assessed heterogeneity using Cochran’s Q test and I^2^ statistics, and Tau-square measures and stratified into low, moderate, and high heterogeneity. Publication bias was assessed with funnel-plot analysis for MACE to evaluate the symmetrical distribution of trials with similar weights. We performed leave-one-out sensitivity analyses for the primary composite outcomes because of the heterogeneous definition to ensure that the results were not dependent on a single study. Prespecified *meta*-regression was performed for the endpoints of MACE and TLR to determine the effect of diabetes and duration of follow-up on the pooled estimates and plots were generated. All statistical analyses were performed using R statistical software, version 4.2.2 (R Foundation for Statistical Computing). A p < 0.05 was considered statistically significant.

## 3 Results

### 3.1 Study selection and characteristics

**Fig. 1:**
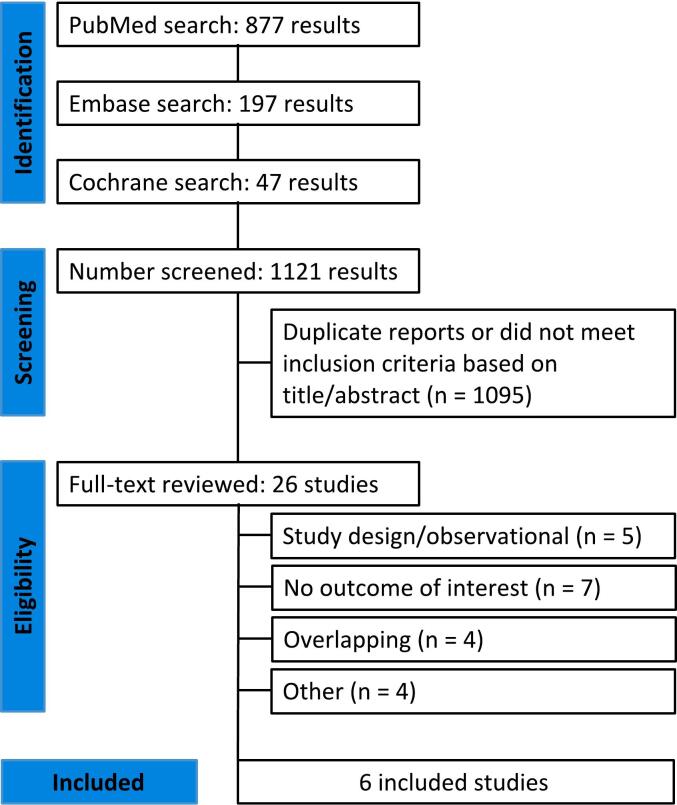
PRISMA flow diagram of study screening and selection.

The initial search strategy yielded 1,121 results. After removing duplicates and studies based on title/abstract, 26 studies were fully reviewed for inclusion and exclusion criteria. Six RCTs [7,[13], [14], [15],^19^,^20^] met the inclusion criteria (Fig. 1).

### 3.2 Study characteristics, quality assessment, and publication bias

A total of 6,290 patients was included, of whom 3,206 (51 %) underwent routine functional stress testing. The weighted mean age of the patients was 63.03 SD10.6 years, with a male predominance of between 68.5–83.0 % in all studies. Drug eluting stents were used in the last three studies [7,13,14]. The follow-up period ranged from 1 to 5 years. The baseline characteristics of the included studies are detailed in Table 1.

### 3.3 TLR

Data on TLR was reported in six studies. The pooled analysis demonstrated the incidence of TLR was nearly twice (2.1 %) in patients who underwent routine functional stress testing compared to those who received standard care (1.4 %) (RR: 1.49; 95 % CI: 1.02–2.18; p = 0.038; I^2^ = 0 %; Fig. 2A).

**Fig. 2A:**
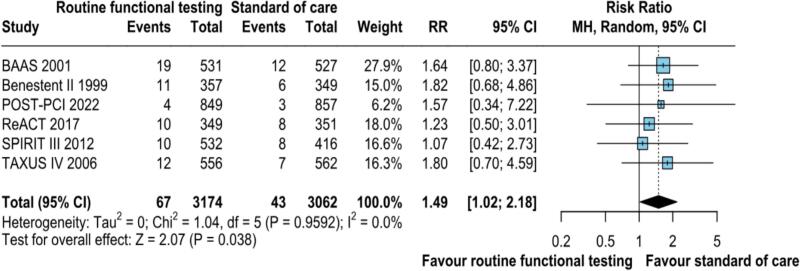
TLR Target lesion revascularisation was significantly higher in the routine functional stress testing compared with the standard of care (p = 0.038). CI, confidence interval, M−H, Mantel-Haenszel, TLR Target lesion revascularisation.

### 3.4 All-cause mortality

All the included RCTs reported all-cause mortality. The pooled analysis demonstrated no significant difference in the risk of all-cause mortality with routine functional stress testing as compared to standard care (RR: 0.89; 95 % CI: 0.48–1.18; p = 0.198; I^2^ = 0 %; Fig. 2B). All-cause mortality occurred in 18 of 3,164 (.57 %) patients that underwent routine functional stress testing. In contrast, 20 (.65 %) all-cause mortality was reported among the 3,058 patients who underwent standard of care.

**Fig. 2B:**
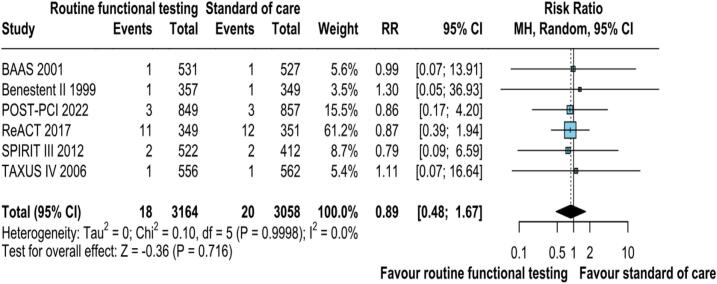
All-cause mortality was not significantly different between the groups. CI, confidence interval, M−H, Mantel-Haenszel.

### 3.5 Myocardial infarction

Data on myocardial infarction was reported in all the 6 studies. The pooled analysis demonstrated no significant reduced risk of MI with routine functional testing compared to standard care (RR: 0.62; 95 % CI: 0.31–1.24; p = 0.174; I2 = 0 %; Fig. 2C). MI occurred in 13 of 3164 (.41 %) patients who had the routine functional stress testing as compared with 20 of 3,058 (.65 %) patients who had standard of care.

**Fig. 2C:**
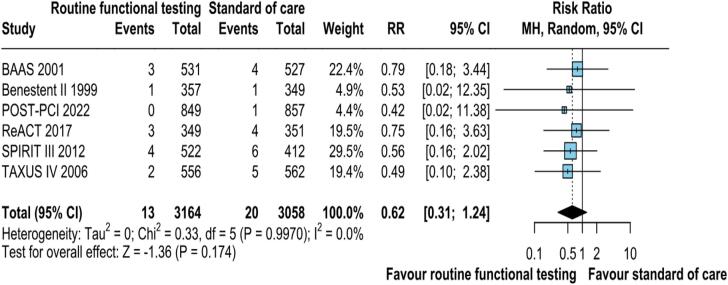
MI was not significantly different between the groups. CI, confidence interval, M−H, Mantel-Haenszel, MI, Myocardial infarction.

### 3.6 Combined all-cause mortality and MI

Data on combined all-cause mortality and MI was reported in all the studies. The pooled analysis demonstrated no statistically significant risk of all-cause mortality and MI with routine functional stress testing compared to standard care (RR: 0.73; 95 % CI: 0.44–1.18; p = 0.198; I2 = 0 %; Fig. 2D).

**Fig. 2D:**
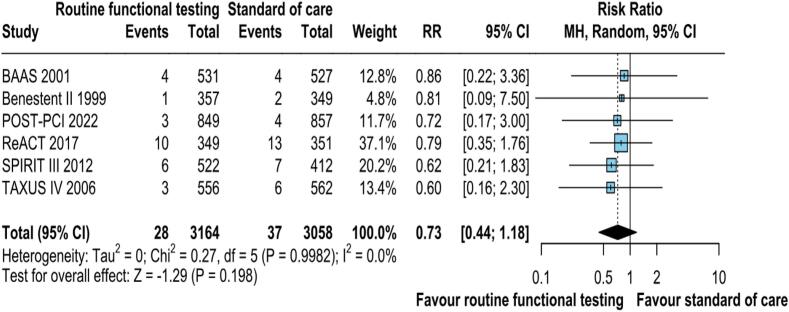
Combined all-cause mortality and MI was not significantly different between the groups. CI, confidence interval, M−H, Mantel-Haenszel, MI, Myocardial infarction.

### 3.7 Hospitalization for any cause

Data on hospitalization for any cause was reported in 2 of the 6 studies. There was no difference in the incidence of hospitalization for any cause between the two groups of surveillance method (RR: 1.22; 95 % CI: 0.24–6.10; p = 0.809; I2 = 0 %; Fig. 2E).

**Fig. 2E:**
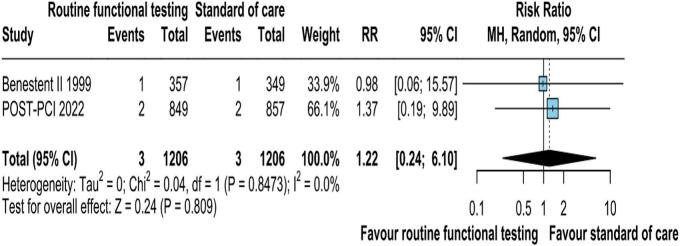
Hospitalisation from any cause was not significantly different between the groups. CI, confidence interval, M−H, Mantel-Haenszel.

### 3.8 MACE

Data on MACE was reported by all the six included studies. The pooled analysis did not demonstrate statistically significant reduced risk of MACE with routine functional stress testing compared to standard care RR: 1.11; 95 % CI: 0.82.1.51; p = 0.480; I2 = 0 %; Fig. 2F).

**Fig. 2F:**
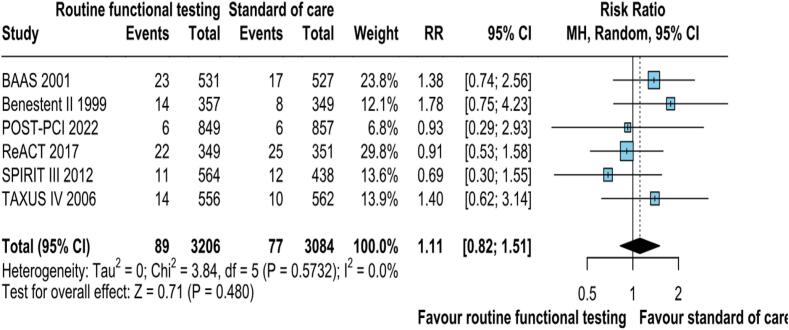
MACE was not significantly different between the groups. CI, confidence interval, M−H, Mantel-Haenszel, MACE, Major adverse cardiovascular events.

### 3.9 Publication bias

All studies were of reasonably high methodological quality. The RoB2 assessment results are shown in Fig. S1. Three studies raised concerns due to single-blind design [15] or sub-randomisation [17,18]. Funnel plot analysis for publication bias of MACE was limited by fewer than ten studies, but no asymmetry or evidence of bias was found (Fig. S2).

### 4.0 Sensitivity, subgroup analyses, and *meta*-regression

Although the included RCTs exhibited low heterogeneity, the leave-one-out analysis, which involved iteratively removing one study at a time, did not affect TLR endpoint (Fig. S3).

There was no difference on subgroup analyses based on the percentage of use of drug-eluting stent for TLR (RR: 1.35; 95 % CI: 0.82–2.24; p = 0.24; I^2^ = 0 %; Fig. S4), MACE (RR: 0.58; 95 % CI: 0.26–1.30; p = 0.19; I^2^ = 0 %; Fig. S5), and all-cause mortality (RR: 0.87; 95 % CI: 0.45–1.68; p = 0.68; I^2^ = 0 %; Fig. S6).

The result of the univariate *meta*-regression carried out for the interactions between TLR and MACE and the covariates of diabetes mellitus and duration of follow-up is shown in Fig. S7-S10. Diabetes (coefficient 0.36; p = 0.55; Fig. S7), follow-up (coefficient 0.52; p = 0.47; Fig. S8) did not significantly modify TLR. Furthermore, diabetes (coefficient 2.05; p = 0.15; Fig. S9) and follow-up (coefficient 1.49; p = 0.23; Fig. S10) did not have significant effect on MACE.

## 4 Discussion

In this updated systematic review and *meta*-analysis of six RCTs and 6,290 patients, we compared routine functional stress testing with the standard of care in high-risk patients after PCI. The main findings from the pooled analysis were as follows: (1) routine functional stress testing was associated with a significantly higher rate of target lesion revascularization than the standard of care, and (2) no significant differences were found between groups for all-cause mortality, myocardial infarction, and MACE.

In symptomatic patients following PCI or coronary artery bypass grafting, abnormal stress test findings have been strongly associated with an increased risk of mortality and MACE. This suggests that functional stress testing remains a valuable tool for risk stratification in individuals with recurrent symptoms after revascularization. However, when applied routinely to asymptomatic patients, stress testing has been shown to primarily lead to higher rates of repeat revascularization without yielding any significant reduction in mortality or MI [15,16]. Importantly, the diagnostic yield of subsequent coronary angiography in these cases does not consistently align with the abnormal stress test results, particularly in asymptomatic individuals. This discrepancy raises concerns about the clinical utility of routine functional stress testing and whether it meaningfully affects long-term outcomes [17].

The POST-PCI trial sought to address these concerns by randomizing 1,706 patients with high-risk anatomical or clinical characteristics who had undergone PCI into two groups: one receiving routine functional stress testing and the other following standard of care. Among the 849 patients in the routine functional stress testing group, there was a 1.0 % reduction in the primary composite endpoint of death from any cause, MI, or hospitalization for unstable angina at two years. However, this reduction was not statistically significant, reinforcing the notion that routine functional stress testing does not necessarily improve clinical outcomes in this population [7]. Further post-hoc analysis in high-risk subgroups, including patients with diabetes, left main coronary artery disease, multivessel disease, or acute coronary syndrome, revealed no significant incremental benefit from routine functional stress testing, suggesting that even among high-risk patients, indiscriminate surveillance strategies may not be warranted [[8], [9], [10]]. These findings are further corroborated by data from the ReACT, SPIRIT III, and TAXUS IV trials, where mandatory follow-up coronary angiography after PCI showed no significant benefits in the primary outcome or incidence of target vessel revascularization [13,14,18]. This suggests that aggressive surveillance may lead to the overdiagnosis of angiographic abnormalities, prompting unnecessary revascularization procedures that do not necessarily translate into better patient outcomes. Given this growing body of evidence, it is unsurprising that recent guidelines from the American College of Cardiology and American Heart Association (ACC/AHA) on revascularization now provide a Class III recommendation against routine stress testing in asymptomatic patients after high-risk PCI. This reflects an evolving consensus that routine functional testing in the absence of symptoms does not improve survival or reduce the risk of MI and may lead to unnecessary interventions. Instead, a more selective, symptom-driven approach is recommended to balance the benefits of early detection with the risk of overtreatment [2].

RCTs in high-risk patients undergoing PCI have consistently reported higher TLR rates without demonstrating a corresponding reduction in the incidence of MI or hospitalizations [6,19,20]. These findings challenge the utility of routine surveillance strategies aimed at detecting restenosis in asymptomatic individuals. In a prior *meta*-analysis, the Benestent II, the Balloon Angioplasty and Anticoagulation Study (BAAS) trials, and planned follow-up angiography were associated with an increased risk of re-intervention. However, the conclusion in this *meta*-analysis and the aggressive approach used in these trials did not translate into a survival benefit for patients, despite a modest reduction in MI rates. It is important to note that the previous *meta*-analysis did not include patients with non-Q-wave myocardial infarction, a subset for which the benefits of routine stress testing followed by coronary angiography remain uncertain [6]. However, these studies were conducted during an era when stent technology was still evolving and the use of dual antiplatelet therapy and high-intensity statins was not yet standard practice [19,20]. Owing to these advancements, contemporary treatment strategies may significantly influence clinical outcomes, potentially altering the relevance of findings from earlier trials.

Consequently, the longstanding perception that systematic stress testing after PCI leads to a reduction in MI rates has persisted, without definitive supporting evidence. To address these uncertainties, a pooled analysis of contemporary RCTs was conducted to clarify the ambiguous conclusions drawn from previous research. Our analysis demonstrated that routine functional stress testing followed by coronary angiography is associated with a higher incidence of TLR in patients with high-risk anatomical or clinical features. However, this is counterintuitive as one might expect routine testing to lead to earlier detection and intervention, potentially reducing the need for revascularization. Furthermore, the routine functional stress testing strategy failed to confer a mortality benefit or reduction in MI, reinforcing the idea that revascularization driven solely by imaging or stress test findings may not always translate into an improved long-term survival. Higher TLR rates imply increased healthcare resource utilisation, potentially leading to higher costs and patient burden, without clear clinical benefits. In addition, the results challenged the assumption that more frequent testing leads to better outcomes, highlighting the need for a careful balance between vigilance and overtreatment. This analysis provides evidence that may influence future guidelines for post-PCI management, potentially leading to more nuanced recommendations.

Therefore, in the absence of clinical deterioration or acute symptoms following PCI, routine functional stress testing is not recommended. Instead, a more selective approach, guided by individual patient risk profiles and symptomatology, may be necessary to avoid unnecessary procedures without compromising patient outcomes.

## 5 Limitations

This study has several limitations. First, the relatively small number of included studies may have reduced the statistical power to detect significant effects, potentially leading to less robust results. A limited sample size increases the risk of type II errors, where true effects may go undetected. Furthermore, while no publication bias was found, studies with negative or non-significant results are less likely to be published, which could have influenced our findings. Second, since the RCTs included in this analysis mainly focused on high-risk patients following PCI, the generalizability of our results to broader patient populations or different risk categories remains uncertain. Patients with lower cardiovascular risk, different comorbidities, or varying clinical presentations may respond differently to the interventions assessed, limiting the applicability of our conclusions. Third, although we performed a leave-one-out sensitivity analysis and subgroup analyses to explore heterogeneity, no significant differences were identified that could affect the applicability of our findings. However, these analyses may not fully account for all unmeasured confounders, and subtle variations in study protocols, patient characteristics, or treatment regimens could still introduce variability in outcomes. Finally, cost-effectiveness was not explicitly analysed in this study. Given the high cost associated with mandatory functional testing followed by coronary angiography, economic considerations are crucial in determining the feasibility and widespread adoption of this approach. Although not encouraged, future studies should incorporate cost-effectiveness analyses to provide valuable insights into the financial implications of these diagnostic strategies, particularly in resource-limited healthcare settings.

## 6 Conclusion

In this *meta*-analysis of RCTs, routine functional stress testing was associated with a higher risk of target lesion revascularization compared to the standard of care. These findings suggest that coronary angiography should be reserved for patients who are symptomatic after PCI.

## 7 Ethical approval

The study was exempt from Human and research ethics committee approval because it uses publicly anonymized data.

## CRediT authorship contribution statement

**Umar G. Adamu:** Conceptualization, Data curation, Formal analysis, Methodology, Software, Visualization, Writing – original draft, Writing – review & editing. **David M. Mashilo:** Supervision, Writing – original draft, Writing – review & editing. **Anupa Patel:** Supervision, Visualization, Writing – original draft, Writing – review & editing. **Nqoba Tsabedze:** Supervision, Writing – original draft, Writing – review & editing.

## Funding

None

## Declaration of competing interest

The authors declare that they have no known competing financial interests or personal relationships that could have appeared to influence the work reported in this paper.
