# Supplementary material for: Routine stress testing with subsequent coronary angiography versus standard of care in high-risk patients after percutaneous coronary intervention: An updated meta-analysis of randomized controlled trials
Source: Int J Cardiol Heart Vasc. 2025 Apr 12;58:101681. doi: 10.1016/j.ijcha.2025.101681 (PMC12017999; doi:10.1016/j.ijcha.2025.101681)
Supplement: Supplementary Data 1 [file mmc1.docx]

**Online Supplementary**

Table S1 : Detailed search strategy used in each database

Table S2: Definition of primary composite outcomes.

Figure S1: RoB2 risk of bias assessment

Figure S2: Funnel plot assessing publication bias of included studies

Figure S3: Leave-one-out analysis of target lesion revascularisation.

Figure S4: Subgroup analysis of studies and percentage of drug eluting stent use and TLR.

Figure S5: Subgroup analysis of studies and percentage of drug eluting stent use and MI.

Figure S6: Subgroup analysis of studies and percentage of drug eluting stent use and all-cause mortality.

Figure S7: Meta-regression of target lesion revascularisation and diabetes

Figure S8: Meta-regression of target lesion revascularisation and duration of follow-up

Figure S9: Meta-regression of major adverse cardiovascular events and diabetes mellitus.

Figure S10: Meta-regression of major adverse cardiovascular events and duration of follow-up

**Table S1.** Detailed search strategy used in each database

| **PubMed** | ("High-risk" OR "high-risk patients" OR "multivessel disease" OR "chronic kidney disease" OR "Renal Insufficiency, Chronic"[Mesh] OR elderly OR "Aged"[Mesh] OR diabetes OR "diabetes mellitus"[Mesh]) AND  ("Percutaneous coronary intervention" OR "Percutaneous Coronary Intervention"[Mesh] OR PCI OR "coronary angioplasty" OR "Angioplasty, Balloon, Coronary"[Mesh] OR "stent implantation") AND  ("functional testing" OR "routine functional testing" OR "stress test" OR "Exercise Test"[Mesh] OR "stress echocardiography" OR "Echocardiography, Stress"[Mesh] OR "myocardial perfusion imaging" OR "diagnostic imaging" OR "diagnostic Imaging"[Mesh]) AND ("Standard Care" OR "Standard of Care"[Mesh] OR "usual care" OR "conservative management" OR "clinical follow-up" OR "symptom-driven") | 877 |
| --- | --- | --- |
| **Cochrane** | ("High-risk" OR "high-risk patients" OR "multivessel disease" OR "chronic kidney disease" OR elderly OR diabetes) AND ("Percutaneous coronary intervention" OR PCI OR "coronary angioplasty" OR "stent implantation") AND ("functional testing" OR "routine functional testing" OR "stress test" OR "Exercise stress testing" OR EST OR "exercise treadmill testing" OR ETT OR "stress echocardiography" OR "myocardial perfusion imaging" OR "diagnostic imaging" OR "cardiac stress testing" OR CST) AND ("Standard Care" OR "usual care" OR "conservative management" OR "clinical follow-up" OR "symptom-driven") | 47 |
| **Embase** | ("High-risk" OR "high-risk patients" OR "multivessel disease" OR "chronic kidney disease" OR elderly OR diabetes) AND ("Percutaneous coronary intervention" OR PCI OR "coronary angioplasty" OR "stent implantation") AND ("functional testing" OR "routine functional testing" OR "stress test" OR "Exercise stress testing" OR EST OR "exercise treadmill testing" OR ETT OR "stress echocardiography" OR "myocardial perfusion imaging" OR "diagnostic imaging" OR "cardiac stress testing" OR CST) AND ("Standard Care" OR "usual care" OR "conservative management" OR "clinical follow-up" OR "symptom-driven") | 197 |

**Table S2.** Definition of primary composite outcomes of MACE

| **Study** | **Primary composite outcomes criteria** |
| --- | --- |
| **Benestent II**  **1999** | Composite of death, myocardial infarction, coronary bypass surgery or repeat repcutaneoues intervention between 6 and 12 moths |
| **BASS**  **2001** | Composite of death, myocardial infarction, and revascularisation at one year |
| **TAXUS IV**  **2006** | Defined as target vessel revascularisation |
| **SPIRIT III**  **2012** | Composite of death, myocardial infarction, and ischaemic -driven revascularisation. |
| **ReACT**  **2017** | Composite of death, myocardial infarction, stroke, emergency hospitalisation for acute coronary syndrome or heart failure. |
| **POST-PCI**  **2022** | Composite of death from any cause, myocardial infarction or hospitalisation for unstable angina. |


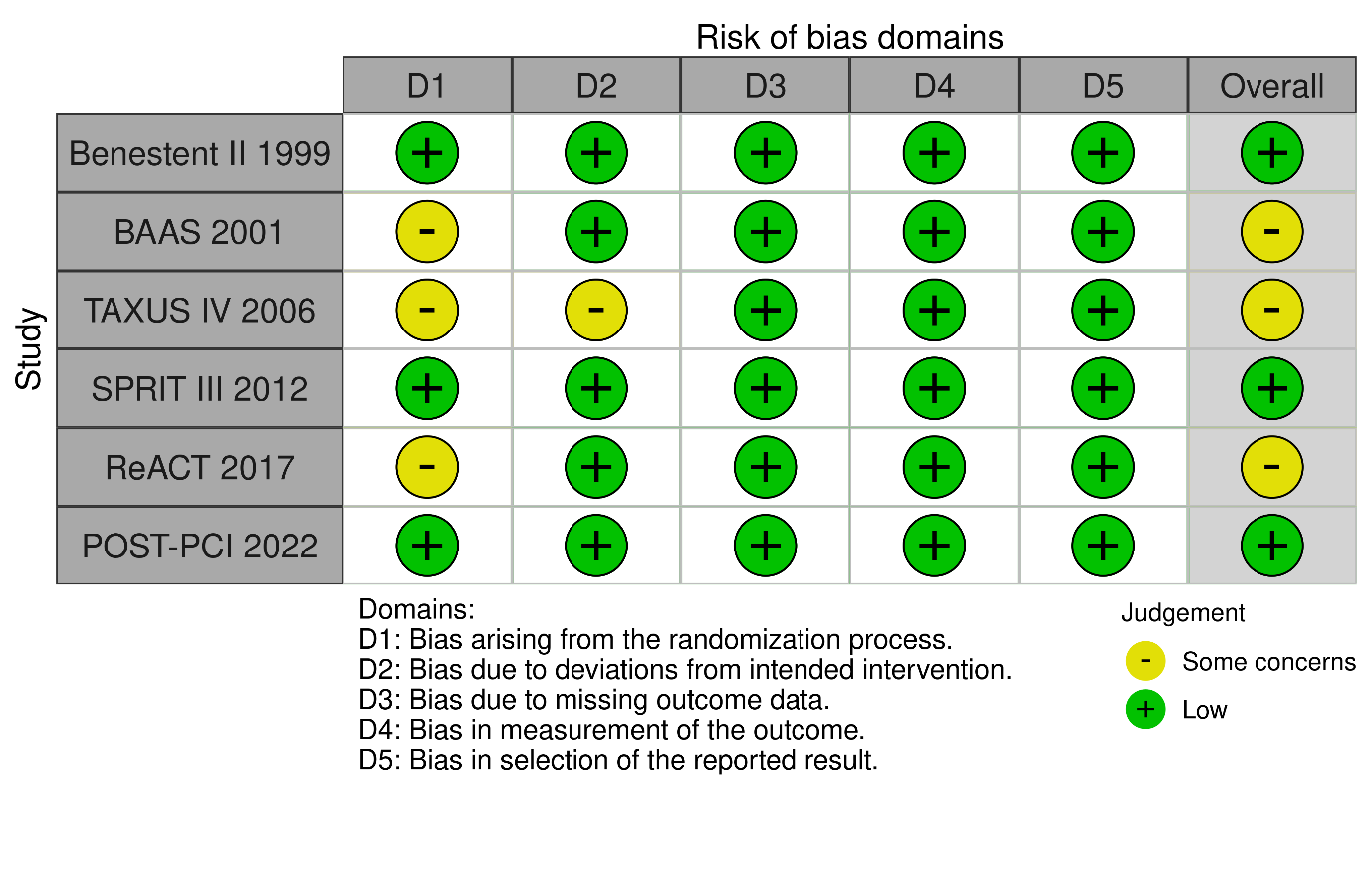


Figure S1. RoB2 risk of bias assessment


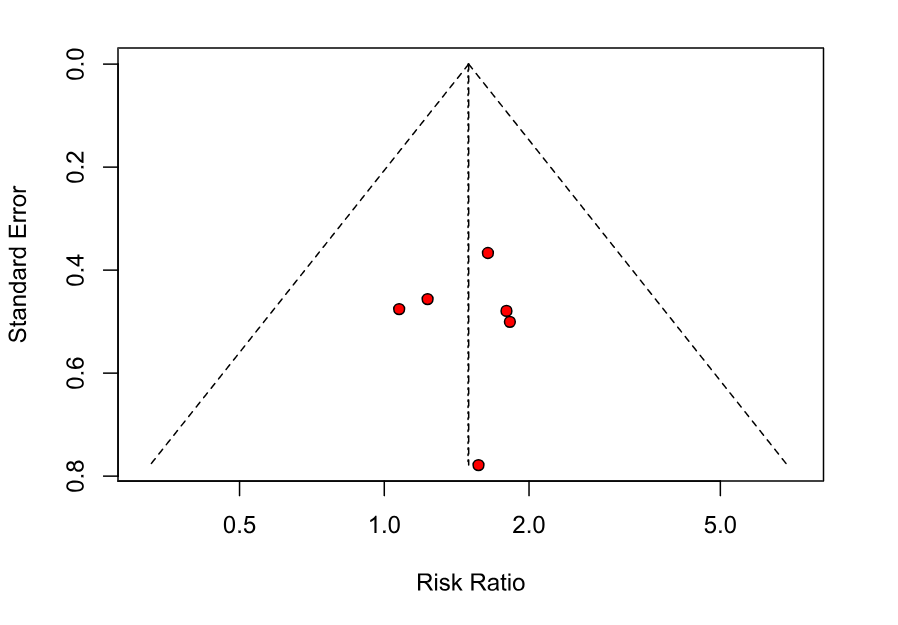


**Figure S2.** Funnel plot assessing publication bias of included studies


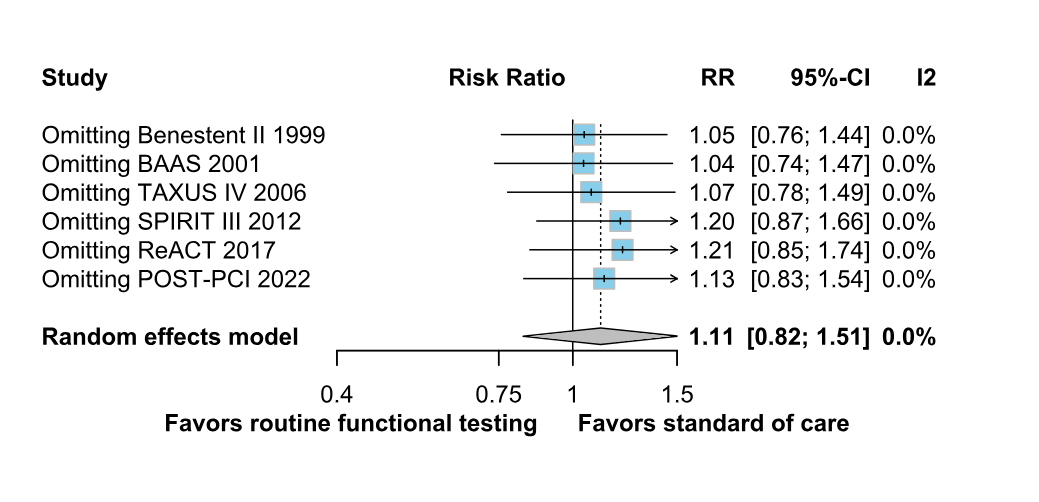


**Figure S3.** Leave-one-out analysis of target lesion revascularisation.


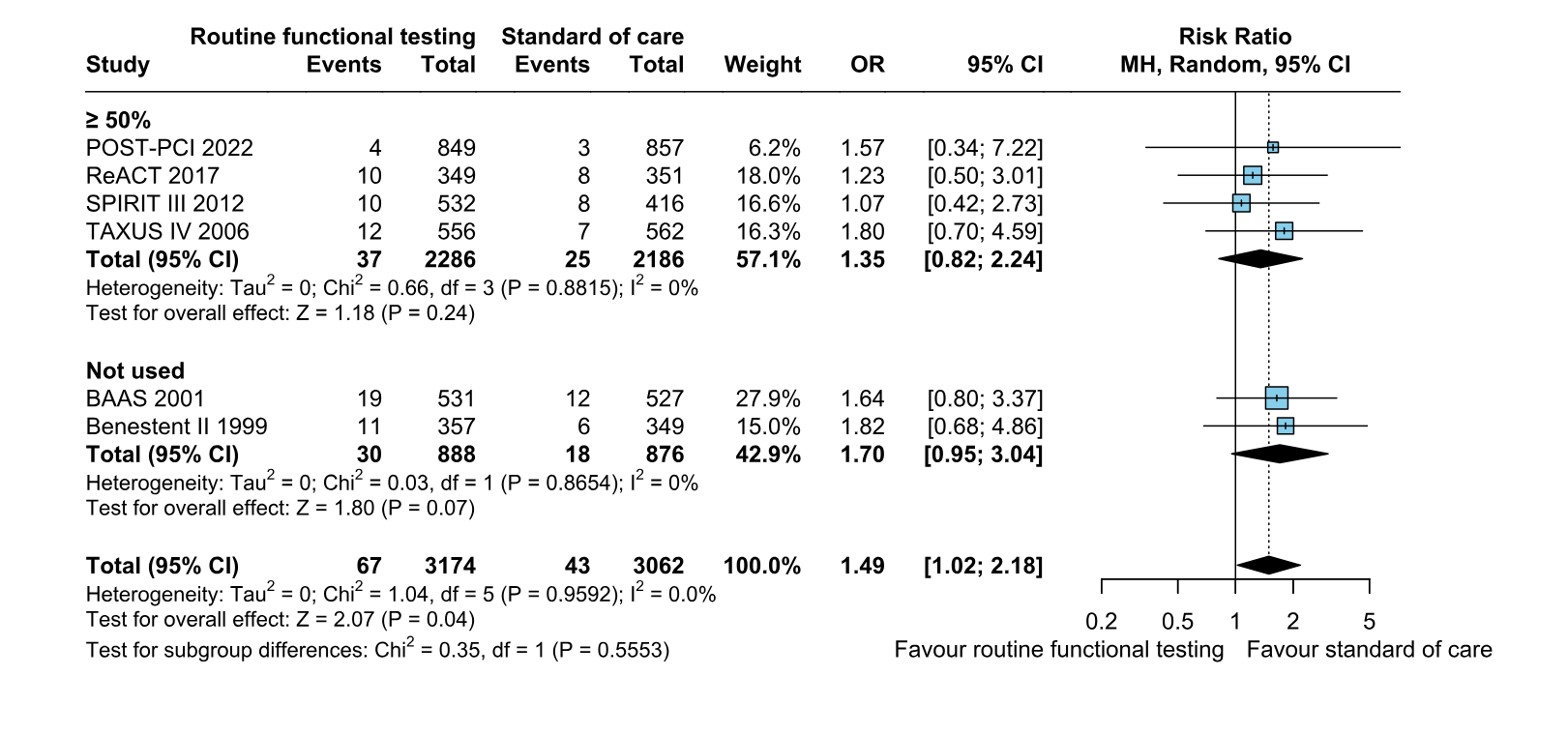


**Figure S4.** TLR: There was a tendency toward significant difference in the incidence of TLR if less than 50% of drug-eluting stent was used (p = 0.07), CI, confidence interval, M-H, Mantel-Haenszel, TLR, Target lesion revascularization


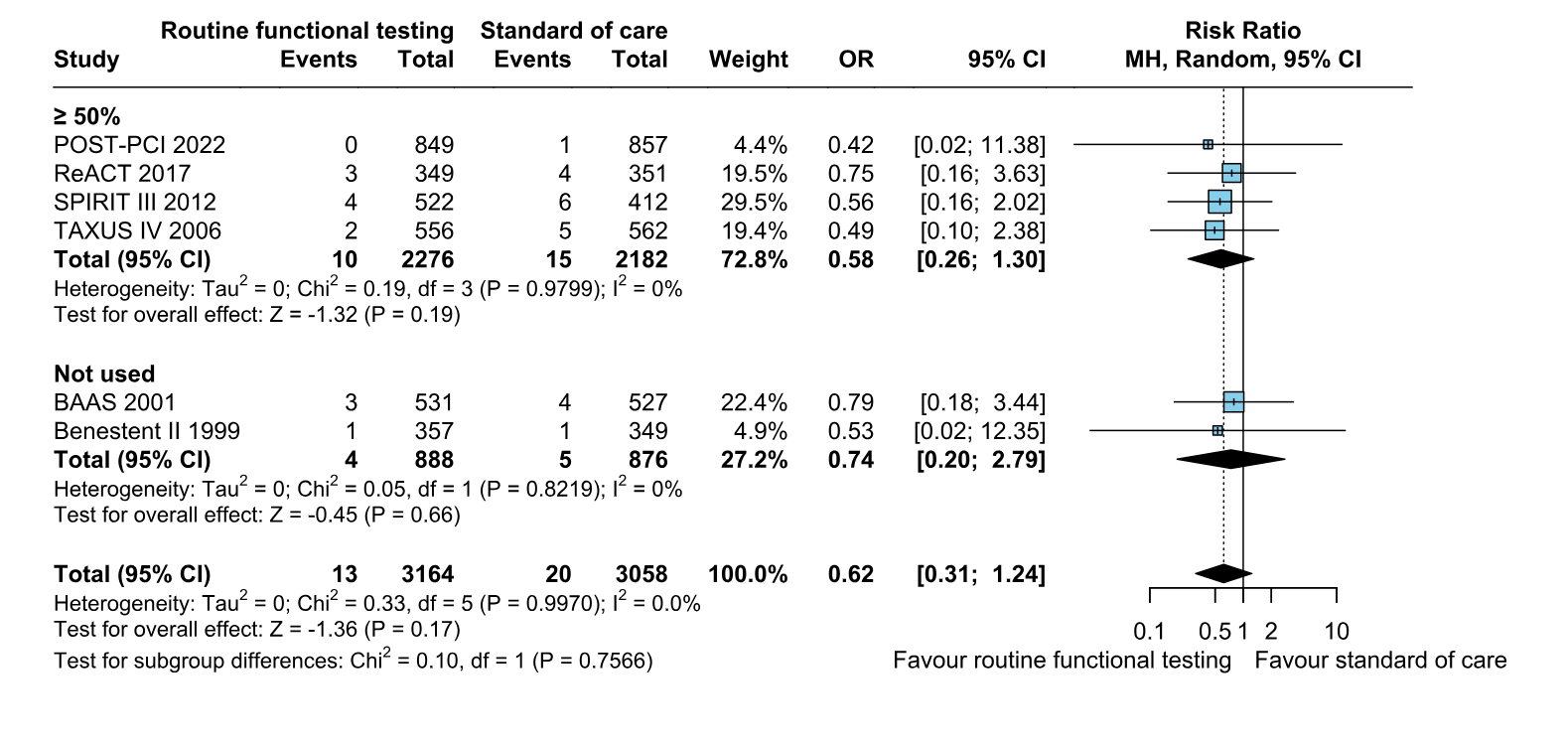


**Figure S5.** MI: There was no significant difference between the percentage of drug-eluting stent use and the incidence of MI between the groups CI, confidence interval, M-H, Mantel-Haenszel, MI, myocardial infarction


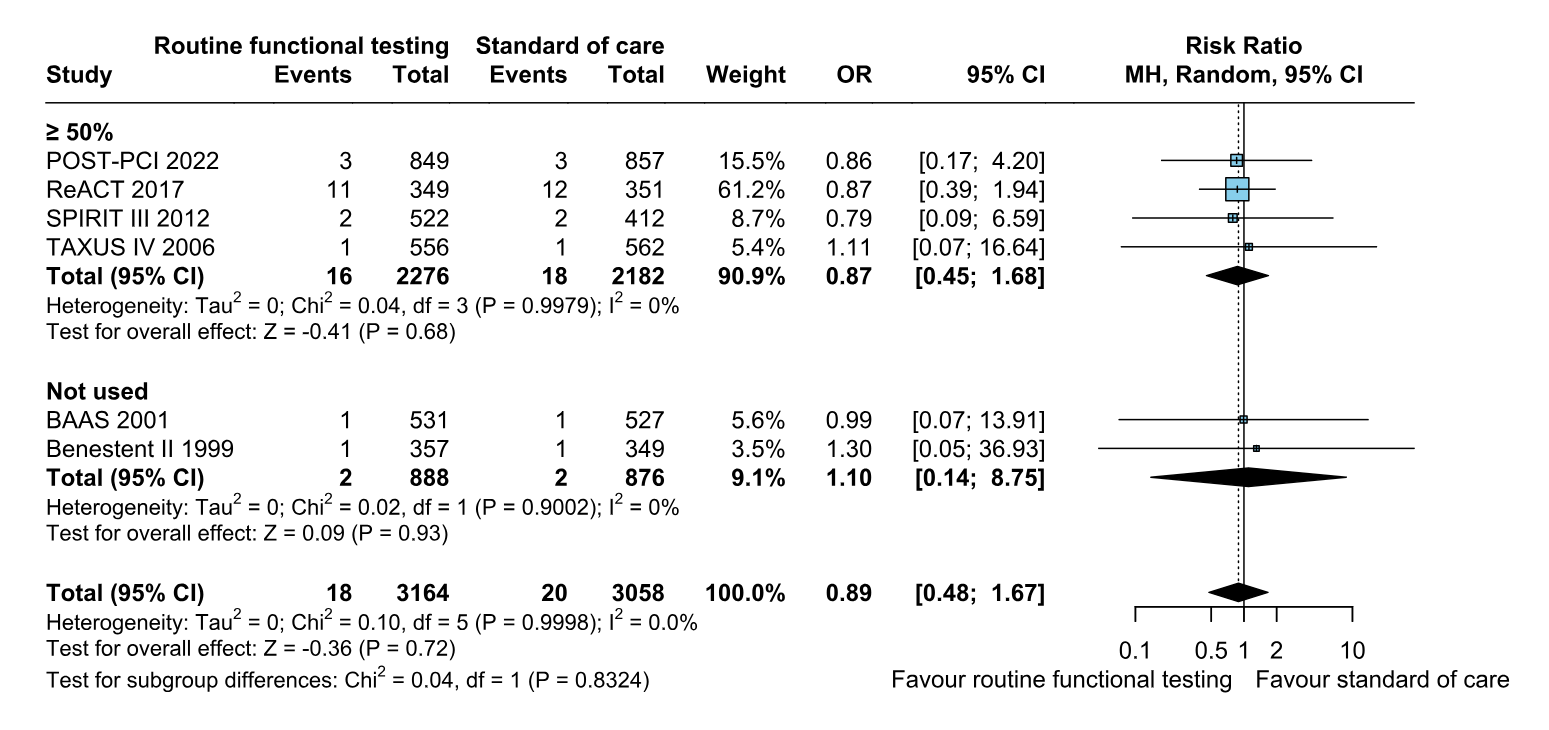


**Figure S6.** All-cause mortality: There was no significant difference between the percentage of drug-eluting stent use and the incidence of all-cause mortality between the groups CI, confidence interval, M-H, Mantel-Haenszel


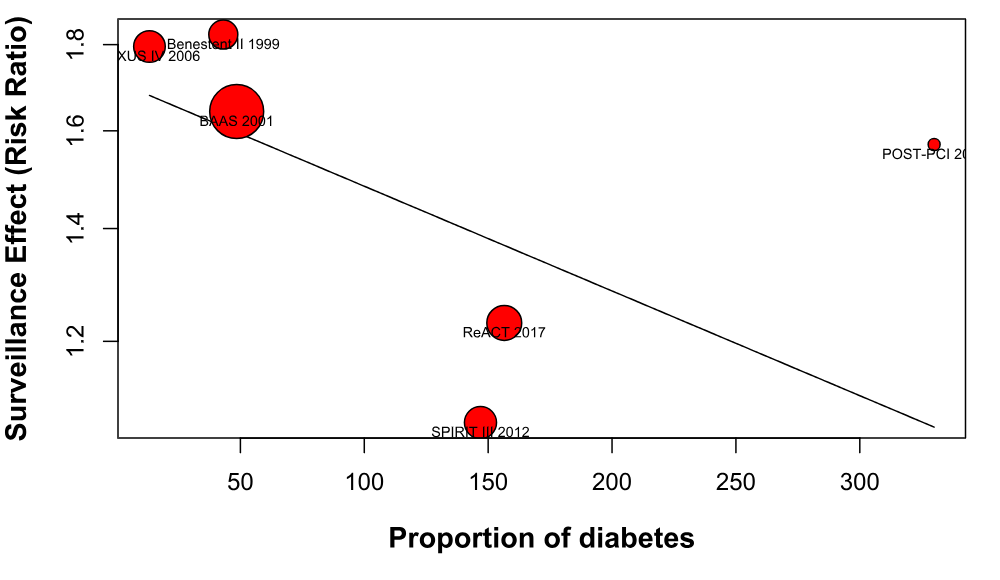


**Figure S7.** Meta-regression of target lesion revascularisation and diabetes mellitus


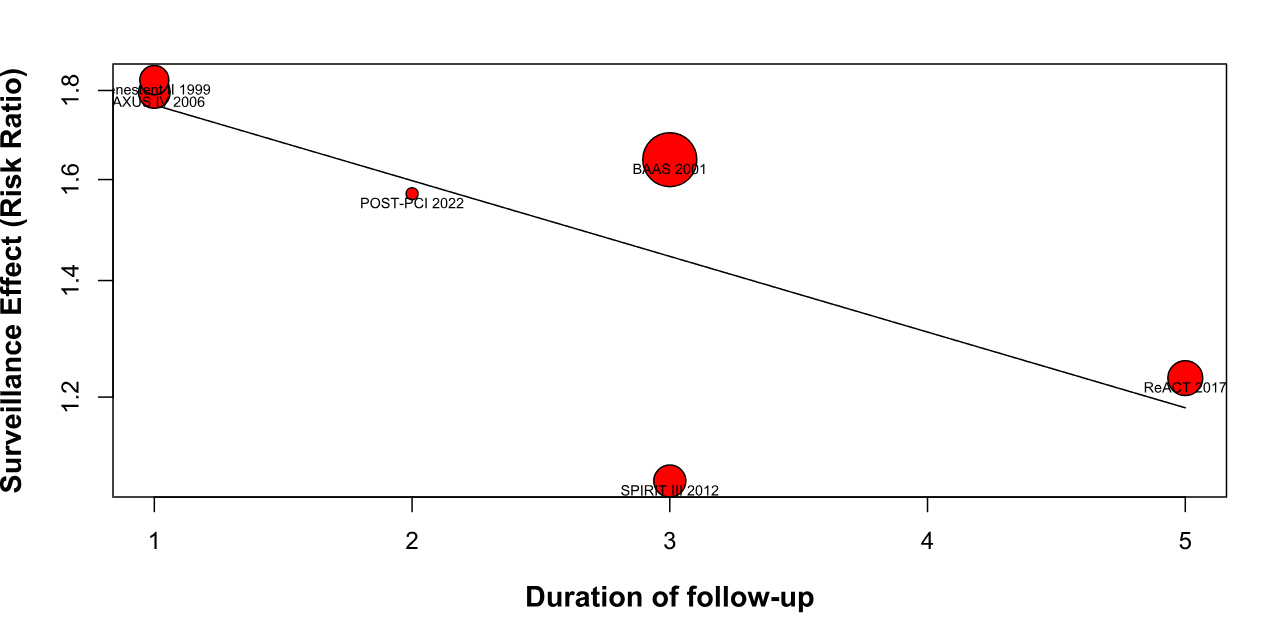


**Figure S8.** Meta-regression of target lesion revascularisation and duration of follow-up


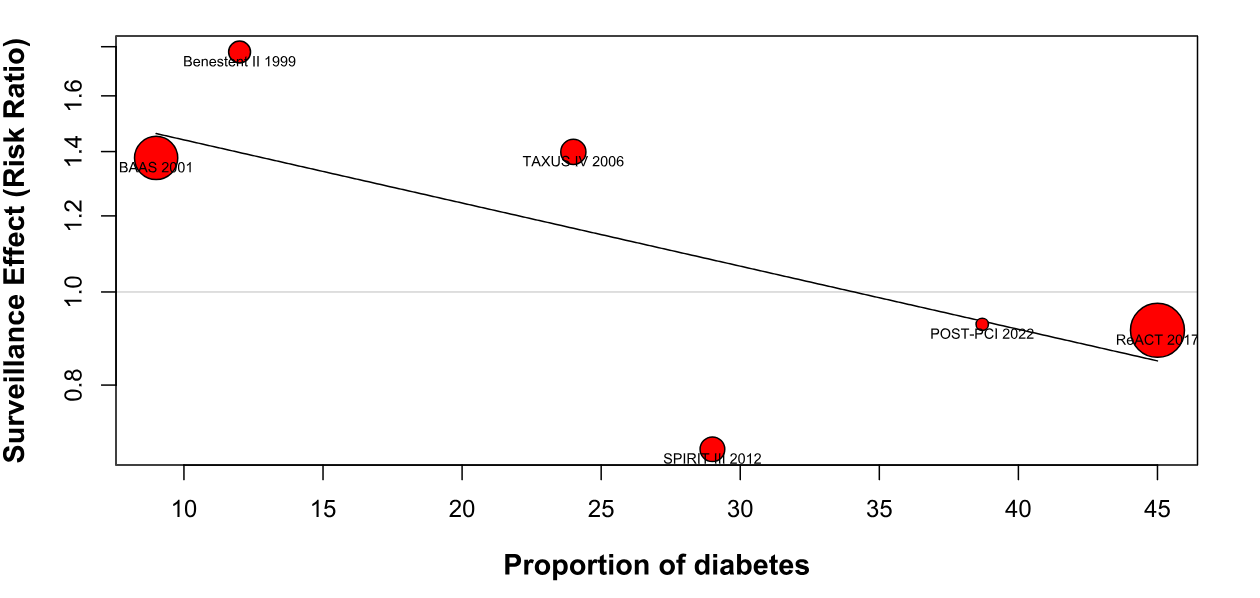


**Figure S9.** Meta-regression of major adverse cardiovascular events and diabetes mellitus


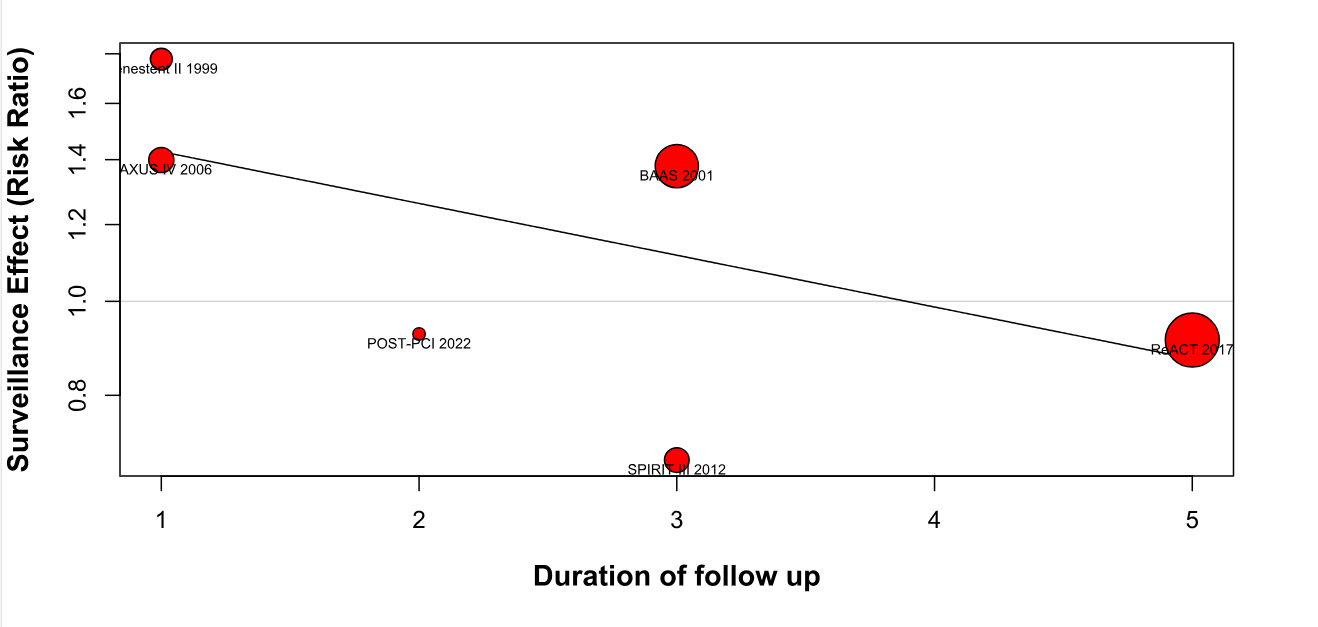


**Figure S10.** Meta-regression of major adverse cardiovascular events and duration of follow-up
